# Comparative study of β-cyclodextrin derivatives with amlodipine inclusion complexes for enhanced solubility, drug release, and anticancer activity

**DOI:** 10.1016/j.ijpx.2025.100368

**Published:** 2025-07-23

**Authors:** Sonaimuthu Mohandoss, Kuppu Sakthi Velu, Naushad Ahmad, Ramachandran Srinivasan, Prasanta Roy, Prathap Somu, Dibyajyoti Haldar

**Affiliations:** a Centre of Molecular Medicine and Diagnostics, Saveetha Dental College and Hospitals, Saveetha Institute of Medical and Technical Sciences, Saveetha University, Chennai, Tamil Nadu, India; b School of Chemical Engineering, Yeungnam University, Gyeongsan 38541, South Korea; c Department of Chemistry, College of Science, King Saud University, P.O. Box 2455, Riyadh 11451, Saudi Arabia; d Centre for Ocean Research, Sathyabama Research Park, Sathyabama Institute of Science and Technology, Chennai 600119, Tamil Nadu, India; e Department of Biotechnology and Chemical Engineering, School of Engineering, Faculty of Science, Technology and Architecture, Manipal University Jaipur, Dehmi Kalan, Jaipur-Ajmer Expressway, Jaipur 303007, Rajasthan, India; f Department of Biotechnology, Manipal Institute of Technology, Manipal Academy of Higher Education (MAHE), Manipal 576104, Karnataka, India

**Keywords:** Cyclodextrins, Amlodipine, Inclusion complex, Solubility, Drug release, Anticancer

## Abstract

Amlodipine (AMD), a calcium channel blocker, has become a viable anticancer treatment because of its biological properties. However, its poor water solubility and low bioavailability hinder its physiological activities and therapeutic applications when administered orally. In this study, inclusion complexes (ICs) of AMD with pure cyclodextrins (CDs) and three different CD derivatives, namely hydroxypropyl β-cyclodextrin (HD), methyl-β-cyclodextrin (MD), and sulfobutylether-β-cyclodextrin (SD), were prepared, and their physicochemical and biological properties were compared. The enhanced solubility of AMD:CD IC formation in aqueous media was measured using UV–Vis and fluorescence spectroscopy, and the binding constants were calculated using the Benesi-Hildebrand method. In addition, phase solubility studies confirmed the formation of 1:1 ICs, which followed an A_L_-type profile. Among the various CD derivatives, AMD:SD exhibited a high apparent stability constant (K_1:1_) of 1447.5 M^−1^, indicating a strong affinity between SD and AMD. The AMD:CDs (1:1) ICs were prepared using the co-precipitation method and characterized to identify the functional groups, crystallinity, morphological changes, and thermal stability, which indicated the successful encapsulation of AMD within CDs. Moreover, molecular docking studies confirmed the encapsulation of AMD within CDs with favorable binding energy and stable interactions. Drug release studies showed an initial burst release followed by a sustained release after 20 min, and the release percentage for the AMD:CDs was between 82 and 98 %. Finally, the AMD:CDs ICs exhibited superior cell viability and cellular uptake in HCT-116 cells using the WST-1 assay compared to that of pure AMD and CDs.

## Graphical abstract

**Figure:**
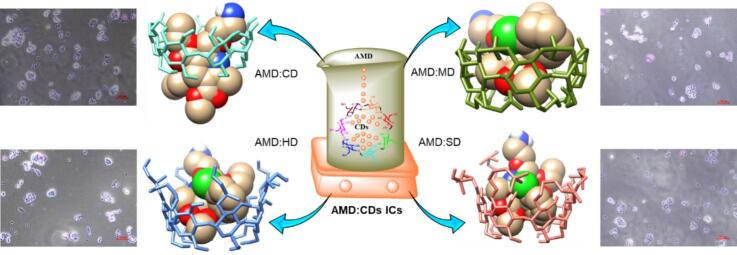

## 1 Introduction

Oral drug delivery is the simplest and most readily available method of drug administration because of its increased stability, lightweight nature, precise dosing, lower production costs, and ease of processing (Lou et al., 2023), and reported improved solubility (Mathew, 2015; Alqahtani et al., 2021). The availability of many existing drugs is impacted by solubility challenges that make it difficult to deliver these new drugs (Kalepu and Nekkanti, 2015). Poorly water-soluble drugs exhibit unstable absorption because their bioavailability is dependent on the drug molecules' dissolution and release profiles, which are crucial factors that directly affect the gastrointestinal tract (Salunke et al., 2022). Several techniques have been reported to improve the dissolving properties of drugs without prior purification via physical and chemical modifications, including particle size reduction (Möschwitzer, 2010), solid dispersion (Mishra et al., 2015), the solvent evaporation method (Iqbal et al., 2015), nanosuspension (Jacob et al., 2020), supercritical fluid processing (Bin et al., 2020), cryogenic techniques (Khan et al., 2022), and inclusion complex (IC) formation (Cheirsilp and Rakmai, 2016). The method selection depends on several factors, including the dosing form, characteristics, and type of additives to be used. Among these factors, the formation of ICs is more readily considered because the drug molecules' physicochemical characteristics, including solubility, thermal stability, melting point, chemical reactivity, and spectroscopic and electrochemical properties, are altered upon IC formation (Jiang et al., 2023; Patil et al., 2010; Kalepu and Nekkanti, 2015; Sanku et al., 2019). The process involves the association of two molecules (host and guest) to form supramolecular ICs through non-covalent interactions, such as hydrogen bonding, van der Waals forces, and hydrophobic and π-π interactions (Abou-Okeil et al., 2018).

Numerous studies over the past two decades have employed cyclodextrins (CDs) as a host molecule with various other guest drug molecules in the pharmaceutical industry. Their unique chemical modification is beneficial in enhancing their solubility and stability by enabling them to create ICs with hydrophobic molecules (Astray et al., 2009; Liu et al., 2021). In general, CDs can be classified as α, β, or γ depending on the number of glucose monomers, such as 6, 7, and 8, respectively. Of the three CDs, β-CD seems to be more useful in various fields, especially in the pharmaceutical and food industries, because of its complex capacity, cavity size, affordability, and increased production rate (Carneiro et al., 2019; Del Valle, 2004). Recently, the preparation of IC products has been used commercially in oral drug formulations, such as nimesulide for gastrointestinal irritation and piroxicam as an anti-inflammatory (Bjarnason and Thjodleifsson, 1999; Brogden et al., 1981). The cyclic oligosaccharides, known as CDs, are composed of D-(+)-glucopyranose units connected by α-1.4 bonds and are produced from starch that is broken down by microbes (Puskás et al., 2023). It has secondary and primary hydroxyl groups; the secondary hydroxyl groups are situated on the wider end of the cone shape, whereas the primary hydroxyl groups are located on the narrower side (Benito and García Fernández, 2019). They may combine with polar or apolar end organic molecules to form a water-soluble complex because of their 3-dimensional structures, which have a hydrophilic exterior and a hydrophobic interior (Hincal et al., 2011; Challa et al., 2005). Several research groups have prepared CD-ICs with various anticancer drug molecules, such as erlotinib (Gontijo et al., 2015), 6-mercaptopurine (Mondal et al., 2022), gemcitabine (Sharma et al., 2023), camptothecin (Fatmi et al., 2015), paclitaxel (Zarrabi and Vossoughi, 2015), silymarin (Ghosh et al., 2011), docetaxel (Ferrati et al., 2015), and liquiritin (Wang et al., 2022), and reported improved solubility and stability, a controlled release profile, and reduced toxicity. Because of CD's low water solubility, various modified CD derivatives, such as methyl-CD (MD), 2-hydroxyethyl-CD (HECD), 2-hydroxypropyl-CD (HD), and sulfobutylether-CD (SD), have garnered interest in recent years for IC formation (Albers and Muller, 1995). Hydroxyl groups of the glucose residues undergo esterification to produce these derivatives. The United States Food and Drug Administration reported that these modified CDs are non-immunogenic, biocompatible, and appropriate for human use (Ferreira et al., 2022). In addition, this particular reaction produces several derivatives with altered properties, such as the potential to transform the solubility of the native crystalline CDs into an amorphous compound (Ferreira et al., 2022; Irie et al., 1988). This change improves solubility when nearby glucose units form intermolecular H-bonds. Our research group has reported that HD CD derivative and SBECD inclusion with guanosine enhanced the solubility and anticancer activity against breast cancer MCF-7 cells (Mohandoss et al., 2019).

Amlodipine (AMD), chemical name 2-[(2-Aminoethoxy)methyl]-4-(2-chlorophenyl)-1,4-dihydro-6-methyl-3,5-pyridinedicarboxylic acid 3-ethyl 5-methyl ester benzenesulfonate is commercially available in tablet form in once-daily dosages of 5 and 10 mg. The oral treatment is readily absorbed and has a bioavailability of approximately 60–65 % (Logoyda, 2020). It acts as a calcium channel blocker and vasodilator to treat angina, high blood pressure, and hypertension as it binds to target receptors in a steady and sustained way, developing a smooth onset of action with 24-h blood pressure regulation (Meredith and Elliott, 1992). AMD typically dissolves in organic solvents but has limited solubility in aqueous buffers; thus, solubility is a crucial factor for the oral administration of this drug (Burges et al., 1989). To enhance the solubility of AMD, Jeevan et al. reported the development of fast-dissolving AMD besylate tablets with co-processed super disintegrants using solvent evaporation methods (Naikwade et al., 2013). The drawback of this process is that the researcher used isopropyl alcohol as an organic solvent. However, these problems could be overcome by considering IC formation between AMD and CDs. Various research groups have reported the preparation of solid ICs using several techniques, including kneading, co-precipitation, solvent evaporation, microwave irradiation, and physical grinding (Sarabia-Vallejo et al., 2023). Among these, co-precipitation methods are preferable because the raw materials are uniformly mixed, the process is low cost, and nanosized particles are produced (Marques, 2010). Other research groups have proposed AMD with CD systems. In 2006, Jadwiga Mielcarek's research group mentioned the inclusion of AMD with MC, reporting that the solid ICs have a molar ratio of 1:1. From an experimental perspective, they explained that the aromatic ring and carbonyl groups in the ester bonds participated in the formation of the ICs (Mielcarek et al., 2006). The supramolecular IC of AMD with CD was established via the kneading, freeze-drying, physical mixture, and co-precipitation methods by Otillia Brada's research group, which reported an increase in drug stability and bioavailability (Bradea et al., 2012). Malakzadeh and Alizadeh (2018) analyzed the comparisons of ICs of AMD with CD and AMD with γ-CD; the solubility was enhanced in both ICs; however, the AMD with γ-CD ICs produced a better antioxidant because of their stronger binding effectiveness (Malakzadeh and Alizadeh, 2018). A superior release profile was achieved in the case of modified CD ICs with poorly water-soluble drug molecules because modified CDs are more water-soluble than pure CDs. In 2022, Novac et al. created ICs of AMD with HC and MC and detailed their physical and chemical characteristics using Fourier transform infrared spectroscopy (FTIR), X-ray diffraction (XRD), and differential scanning calorimetry analyses. Our primary focus was to enhance the anticancer efficacy of AMD treatment. The limited solubility and stability of AMD restrict its effectiveness, which is why we developed a supramolecular complex of AMD:CDs to improve its performance (Novac et al., 2022). Nanda research group reported the generation of a film similar to those made from AMD and CD-based materials. This film incorporated hydroxypropyl methylcellulose (MPMC) along with AMD and various CDs (β-CD, HPβ-CD, and Sβ-CD) and demonstrated an enhanced dissolution and release profile, particularly for the films containing Sβ-CD. This improvement can be attributed to the high dissolution rate and amorphization (Nanda et al., 2018). Although similar studies have been previously conducted, none have reported their anticancer effects on HCT-116 cells. This study aimed to address this gap in the available literature.

Despite previous reports on the inclusion complexes of amlodipine (AMD) with individual cyclodextrins, our study presents a comprehensive and comparative analysis involving native β-cyclodextrin (CD) and three chemically modified derivatives; hydroxypropyl-β-cyclodextrin (HD), methyl-β-cyclodextrin (MD), and sulfobutylether-β-cyclodextrin (SD) for the first time in solid-state form. Importantly, the AMD:SD inclusion complex has not been previously reported, particularly in the context of anticancer applications. Our research uniquely combines advanced physicochemical and structural characterization techniques, including UV–Vis and fluorescence spectroscopy (Benesi–Hildebrand analysis), FTIR, XRD, SEM, TGA, and molecular docking simulations to confirm complex formation, stability, and interaction energetics. In addition, the phase solubility studies indicated the formation of 1:1 ICs with an A_L_-type profile, and AMD:SD exhibited the highest stability constant (K_1:1_ = 1447.5 M^−1^). Moreover, our investigation beyond structural analysis by conducting in vitro drug release studies in PBS (pH 7.4), revealing a sustained release profile of up to 98 % for AMD:SD. Significantly, the anticancer efficacy of these complexes against HCT-116 human colorectal cancer cells showing that AMD:SD displays superior cytotoxic activity (90 % inhibition at 70 μg/mL) compared to other complexes and pure AMD. These integrated analytical and biological findings provide new insights into the enhanced performance and therapeutic potential of AMD when formulated with modified CDs, particularly SD. Therefore, this study as a novel and promising approach for improving the solubility, bioavailability, and anticancer application of poorly water-soluble drugs.

## 2 Materials and methodology

### 2.1 Materials

Amlodipine (AMD, purity 98 %) was obtained from Sigma-Aldrich, β-Cyclodextrin (CD), 2-hydroxypropyl β-Cyclodextrin (HD), methylated β-Cyclodextrin (MD) and sulfobutylether-β-(SD) Cyclodextrin (SD) were purchased from TCI Chemicals and used without any prior purification. The remaining chemicals and reagents were standard in laboratory grade obtained from TCI. All experimental procedures were carried out with de-ionized water, purified using a Milli-Q system.

### 2.2 Preparation of inclusion complex (ICs) in aqueous medium

At first, the different concentrations (0, 0.002, 0.004, 0.006, 0.008. 0.01, and 0.012 M) of CDs (CD, MD, HD, and SD) The pristine CDs of CD exhibit. The AMD concentration was fixed at 0.001 M (4.08 mg in 100 mL) with 10 mL of ethanol. Then, 10 μL of this solution was added to each concentrated CD solution. The resulting mixtures were shaken *v*igorously and allowed to stand for 5 min at room temperature (RT) to reach equilibrium. All measurements were performed in triplicate.

### 2.3 Preparation of inclusion complex (ICs) in solid state

The simple and cost-effective co-precipitation method was used to prepare the solid ICs (Jiang et al., 2019). In brief, CD, MD, HD, and SD (1.134 g, 1.310 g, 1.541 g, and 1.134 g, respectively) were each placed in separate 100 mL beakers. AMD (4.0 mg) was then added to each beaker and the mixtures were dissolved in an ethanol:water system (1:1.5, *v*/v) maintaining a host:guest molar ratio of 1:1. The solutions were stirred overnight at room temperature and subsequently heated to 70 °C, leading to the formation of a precipitate. After filtering, the precipitated ICs were recovered and washed with the minimum amount of ethanol and water to eliminate the uncomplexed or unreacted AMD. Then, the mixture was dried in a vacuum at RT for two days and kept in an airtight container.

### 2.4 Determination of binding constant

The Benesi–Hildebrand plot was used to calculate the binding constant for the prepared aqueous ICs in AMD:CD, AMD:HD, AMD:MD and AMD:SD by ultraviolet and fluorescence spectroscopy measurement (Yang et al., 2000). In brief, the aqueous ICs (different concentrations of CDs with AMD) solutions were used in this absorption and emission measurement. All solutions were allowed to equilibrium for over 5 min at RT, in the FL measurements the slit width and step size for the instrumental measurements were 5 nm and 1 nm, respectively. Each experiment was run 3 times at RT. The binding constants of the inclusion complexes (ICs) were determined using the Benesi–Hildebrand equations as follows (Scott, 1956).

(1) $$\frac{1}{\left(A-A_{0}\right)}=\frac{1}{\left(A^{\prime }-A_{0}\right)}+\frac{1}{k\left(A^{\prime }-A_{0}\right)}\;\left[\beta -\mathrm{CDs}\right]\ldots \ldots ..$$

Where A_0_ is the intensity of absorbance without CDs, A is the absorbance of individual concentration of CD, A′ is the maximum concentration of CD and *k* is the binding constant.

(2) $$\frac{1}{\left(F-F_{0}\right)}=\frac{1}{\left(F^{\prime }-F_{0}\right)}+\frac{1}{k\left(F^{\prime }-F_{0}\right)}\;\left[\beta -\mathrm{CDs}\right]\ldots \ldots ..$$

In the above eq. (2), the F_0_ emission intensity of the guest molecule without CDs, F, is the particular concentration of CD, F′ is the maximum concentration of CD, and *k* is the binding constant. In the thermodynamic parameter of enthalpy, ΔG was also determined from the binding constant “K” value (Belay, 2012).

(3) ΔG=−RTlnK……..

Where R is the Ideal Gas Constant (0.008314 kJ/mol.K), T is the temperature of these reactions (298 K), and K is the binding constant values of the ICs.

### 2.5 Phase solubility study

The solubility studies of ICs (AMD:CD, AMD:HD, AMD:MD and AMD:SD) were carried out using the methodology described by Higuchi and Connors (Nicolescu et al., 2010). In brief, the excess amount of AMD was added to the different concentrations of CDs solution at a level is 0.002 to 0.012 M. All the solutions were prepared in a 25 mL volumetric flask that was shaken on a shaker set to 250 rpm/min for 24 h at RT. Afterward, the samples were stored in a dark room for 24 h to allow the equilibrium process, followed by filtration using Whatman filter paper. The filtrate was placed into the UV-Spectroscopy by measuring the absorbance 365 nm. In addition, three consecutive samples were used to determine the same amount of AMD after the mixture was shaken. The stability constant was determined using these experiments by following the equation,

(4) $$K_{s}=\frac{\text{Slope}}{S_{0}\left(1-\text{Slope}\right)}\ldots \ldots ..\ldots ..$$

### 2.6 Characterizations

The absorbance and fluorescence properties of AMD and their inclusion complex formation of the AMD:CDs were investigated using Ultraviolet-Visible (UV–Vis) spectroscopy UV 3220 (Optizen) and fluorescence spectra (FL; Shimadzu spectrofluorometer, RF-5301). The functional groups and chemical composition of the pure molecules and AMD:CDs ICs were characterized by fourier transform infrared spectroscopy (FTIR, Perkin Elmer). FTIR spectra were recorded with a resolution of 4 cm^−1^, with 64 scans taken in the wavenumber range of 4000–400 cm^−1^. The crystallinity of the pure molecules and AMD:CDs ICs was examined using X-ray diffraction (XRD, PANalytical X'Pert) with Cu Kα radiation. XRD data were collected in the 2θ range of 10°–80°, providing insight into the crystalline structure of the samples. The morphology of AMD and its inclusion complex formation of the AMD:CDs was examined using scanning electron microscopy (SEM, Carl Zeiss SMT, Oberkochen, Germany). Thermal characteristics of pure molecules and AMD:CDs ICs were assessed by Thermogravimetric Analysis (TGA, TA Instruments, New Castle, DE, USA). The samples were heated in a nitrogen atmosphere from 25 °C to 400 °C at a heating rate of 20 °C per minute to study their thermal stability and decomposition behavior.

### 2.7 In vitro releasing studies

To assess the release profile of the ICs at various time intervals, a UV spectrometer (UV 3220 Optizen) operating in the wavelength range of 200–800 nm. In brief, 10 mg of each complex (AMD:CD, AMD:HD, AMD:MD, and AMD:SD) along with pristine AMD was dissolved in 10 mL of phosphate buffer saline (PBS, pH ∼ 7.4) and placed in a 100 mL beaker. The entire phosphate buffer saline with AMD:CDs solution was continually stirred at 10-min intervals, and 2 mL phosphate buffer saline solution was collected simultaneously with the addition of the freshly prepared phosphate buffer saline solution (2 mL) to the mixture unit of time. The calibration curve was established by plotting the concentration of the ICs against their absorbance. Additionally, a graph was plotted to show the cumulative release of AMD over time. The cumulative release was calculated using the following equation (Ye et al., 2015);

(5) $$\text{Cumulative release}\;\left(\%\right)=\frac{\text{Amount of}\;\mathrm{AMD}\;\text{released from the}\;\mathrm{ICs}\;}{\text{Totral amount of}\;\mathrm{AMD}\;\text{released from the}\;\mathrm{ICs}\;}\times 100\;$$

### 2.8 Anticancer studies

The human colorectal cancer cell line HCT116 was cultured in Dulbecco's Modified Eagle Medium (DMEM) supplemented with 10 % fetal bovine serum and 100 mL of penicillin-streptomycin, maintained at 37 °C in a humidified atmosphere containing 5 % CO_2_. For the cytotoxicity assessment using the MTT WST assay, 1 × 10^4^ cells per well were seeded in 96-well plates and incubated overnight to allow adherence. The cells were then treated with various concentrations (0–70 μg/mL) of inclusion complexes (ICs) of AMD with different CDs: CD, HD, MD, and SD. To serve as controls, pristine AMD and each CDs alone (CD, HD, MD, SD) were also tested for comparative evaluation of cytotoxic effects. After treatment, 0.5 mg/mL of WST dye reagent was added to each well, and the plates were incubated in the dark at room temperature for 2 h. Subsequently, 100 μL of dimethyl sulfoxide (DMSO) was added to dissolve the purple formazan crystals formed by viable cells. The absorbance was measured at 570 nm using a microplate reader, and cell viability was calculated accordingly to determine the cytotoxic potential of the tested compounds (Veiga et al., 2024).

(6) $$\text{Cell viability}\;\left(\%\right)=\frac{\text{Absorbance sample}\;}{\text{Blank absorbance}\;}\times 100\;$$

## 3 Results and discussion

### 3.1 UV–Vis and fluorescence spectroscopy

**Fig. 1:**
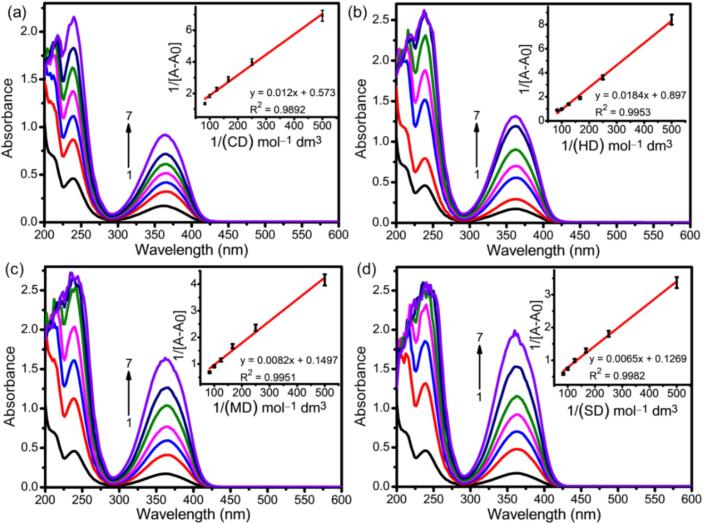
Absorption spectra of AMD in different (a) CD, (b) HD, (c) MD, and (d) SD concentrations (mM): (1) 0, (2) 0.002, (3) 0.004, (4) 0.006, (5) 0.008, (6) 0.010 and (7) 0.012. Inset; B—H plots for formation of (1:1) 1/(A − A_0_) vs. (a) 1/[CD], (b) 1/[HD], (c) 1/[MD], and (d) 1/[SD].

The absorbance spectra of the pure AMD and AMD with the addition of various CDs (CD, MD, HD, and SD) solutions at different concentrations (0–0.012 M) are shown in Fig. 1. The pure AMD exhibited two absorption bands, 235 and 365 nm, due to the presence of the π-π^⁎^ transition of the aromatic group and pyridine derivatives (Safila et al., 2014). With the addition of CDs to AMD, the absorbance increased, and the peaks exhibited a bathochromic shift (from 237 to 230 nm), which suggests the formation of ICs such as AMD:CD, AMD:HD, AMD:MD, and AMD:SD. The hydrophobic interaction between AMD and the CDs is exploited by this stance. The binding constant (K) and stoichiometry (1:1) of the non-bonding interaction were determined mathematically using the Benesi-Hildebrand (B—H) technique. This approach acquired the absorbance and emission data as a combination of the host, guest, and host–guest complex. The B—H graph (inserted within the UV graph) is plotted as 1/(A-A_0_) vs. 1/CDs, and a linear correction coefficient was obtained. The binding constant values are 48.30, 84.27, 88.70, and 110.76 M^−1^ for the ICs of AMD:CD, AMD:HD, AMD:MD, and AMD:SD, respectively. Moreover, the linearity observed in the B—H plot between 1/(A-A_0_) and 1/CDs reveals a 1:1 M stoichiometry between AMD and CDs, which indicates that each CD molecule binds to a single AMD molecule. Moreover, obtained Gibbs free energy values are 9.76, 11.16, 11.29, and − 11.85 kJ/mol (Table S-1 to S-4). The negative values of ΔG indicate the spontaneous ICs formation at room temperature.

**Fig. 2:**
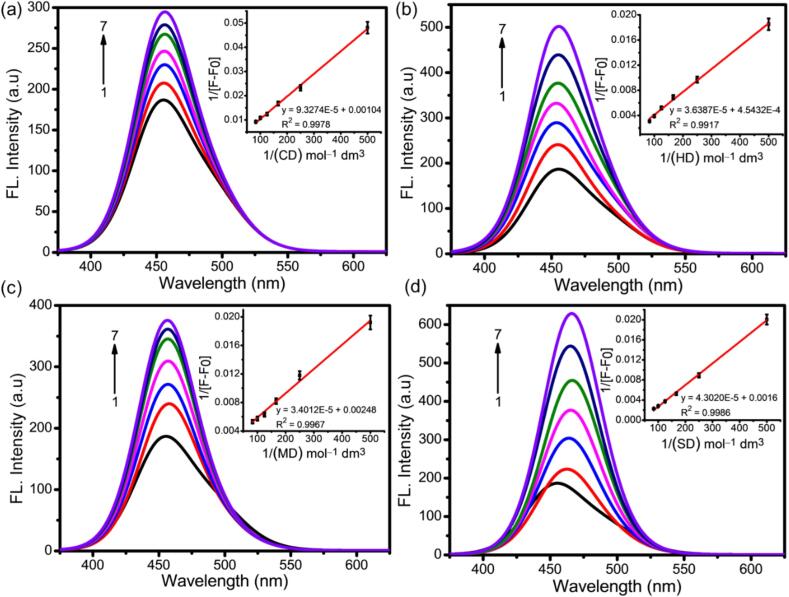
Fluorescence spectra of AMD in different (a) CD, (b) HD, (c) MD, and (d) SD concentrations (mM): (1) 0, (2) 0.002, (3) 0.004, (4) 0.006, (5) 0.008, (6) 0.010 and (7) 0.012. Inset; B—H plots for formation of (1:1) 1/(F − F_0_) vs. (a) 1/[CD], (b) 1/[HD], (c) 1/[MD], and (d) 1/[SD].

The fluorescence spectra of pure AMD and the addition of increasing concentrations of CD solutions are shown in Fig. 2. An emission peak was seen for pure AMD at 463 nm with excitation at 365 nm, while the emission intensities increased with the addition of increasing concentrations of CD solutions, and the emission peak shifted to a higher wavelength (463 to 465, 463 to 467, 463 to 468, and 463 to 475 for ICs of AMD:CD, AMD:HD, AMD:MD, and AMD:SD, respectively) (Saputri et al., 2022). The B—H plot of 1/(I—I_0_) vs. 1/[CDs] is inserted in all the fluorescence graphs, and it is a linear plot, R^2^ < 0.99, thus confirming that the complexation between the AMD:CDs and the binding constant (Ka) values were calculated as 52.74, 87.42, 99.99, and 155.40 M^−1^. In addition, the B—H linear graph verified the 1:1 stoichiometry formation between AMD and each CD, which is essential for improving the solubility and stability of AMD following complexation. Moreover, the calculated Gibbs free energy values were 9.98, 11.26, 11.61, and 12.71 kJ/mol for the ICs of AMD:CD, AMD:HD, AMD:MD, and AMD:SD, respectively (Table 1–4). The increasing order of the binding constant of ICs is as follows: AMD:CD < AMD:HD < AMD:MD < AMD:SD. With the improved hydrophobicity and hydrogen bonding possibilities provided by the hydroxyl groups, the CD frequency shows greater binding constants in the ICs than in the non-hydroxylated pure CD. In the instance of SD, the binding strength was higher than that of HD and MD because the substitution of sulfobutyl ether for hydroxyl groups increased the overall hydrophobicity of SD (Papezhuk et al., 2020). Therefore, AMD:SD had a higher binding constant (UV–Vis and fluorescence B—H plots) than the other ICs, indicating the establishment of a stronger and more stable host–guest interaction (Evrard et al., 2002). This suggests that the IC is less likely to dissociate as the host molecule of SD and the guest molecule of AMD are firmly linked together. In previous studies, sulfated CDs, in comparison with their non-sulfated counterparts, generally displayed an enhanced binding constant (Chen et al., 2001) and ICs due to the insertion of negatively charged sulfate groups, which strengthen electrostatic interactions with positively charged guest molecules. Overall, these findings suggest that AMD is appropriate for ICs formation with all evaluated CDs, with a 1:1 stoichiometric ratio and high binding constant values. Therefore, further examination of ICs for phase solubility studies is recommended, as well as the preparation of solid ICs, because solid ICs are more suitable in oral drug delivery systems. This is because they offer higher thermal and physical stability than liquid ICs, which is crucial for the formulation of stable and soluble drugs.

### 3.2 Phase solubility test

**Fig. 3:**
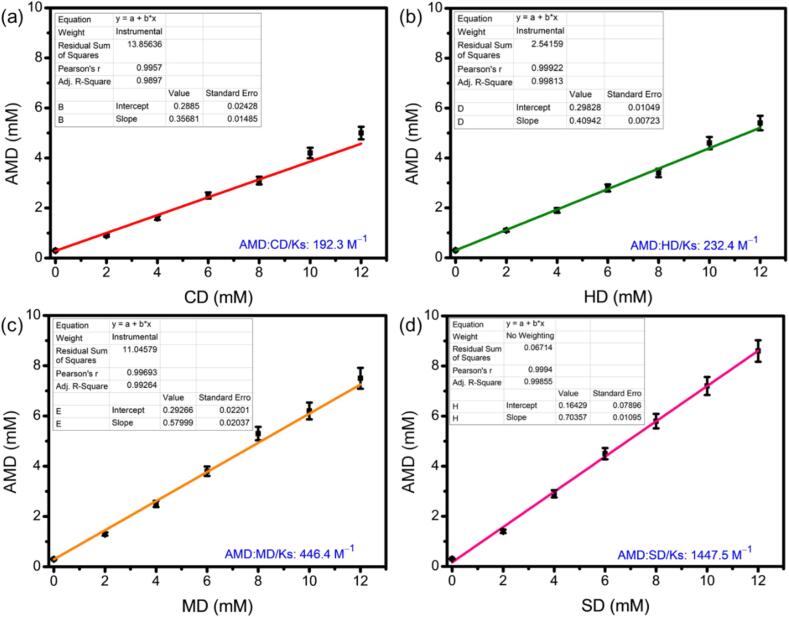
Phase solubility diagram of IC of (a) AMD:CD, (b) AMD:HD, (c) AMD:MD and (d) AMD:SD in water (*n* = 3).

Phase solubility studies are useful for determining the solubility rate of prepared ICs, and the apparent solubility constant was calculated. The ICs were prepared using various CD molecules, such as CD, HD, MD, and SD, to address the solubility issue of AMD. The interaction between AMD and CDs was evaluated using phase solubility studies in a water medium. Subsequently, the phase solubility profile was developed by measuring the total amount of AMD dissolved against the increasing concentration of CDs (0–0.012 mM). According to Higuchi and Connors, the relationships were linear, indicating an A_L_-type phase solubility profile (Fig. 3). The slope (representing the linear increase in AMD solubility with the increasing CD concentrations) suggests the formation of a 1:1 molecular complex (Mader and Higuchi, 1970; Connors and Higuchi, 1965). Based on these profiles, the binding constants or solubility constants (K_s_) of the complexes were calculated as 192.3, 232.4, 446.4, and 1447.5 M^−1^ for AMD:CD, AMD:HD, AMD:MD, and AMD:SD, respectively. In all CDs, the binding constants for AMD exhibited the increasing order of AMD:CD < AMD:HD < AMD:MD < AMD:SD. A similar order was obtained in correlation with our B—H plots. This result indicates that the binding and solubility improvement are dependent on the CD substitution and increased hydrophilicity (Saokham et al., 2018).

Furthermore, SD was a superior solubilizer compared to native CD and was the most effective solubilizer of AMD. The results of this study were consistent, indicating that SD is a crucial host molecule for complex formation with anticancer drug molecules. This may be due to several factors, such as the sulfate groups that impart a negative charge to the glucose units of CDs. These negative charges enhance electrostatic interactions, thus contributing to stronger binding with the guest molecules. These strong interactions add an extra layer of stability to the complex compared with non-sulfated CDs, making it less prone to dissociation or degradation under specific conditions (Huang et al., 2024). As a result, the ICs formed with SD is more stable and exhibits stronger binding with hydrophobic guest molecules within the SD cavity.

### 3.3 FTIR analysis

**Fig. 4:**
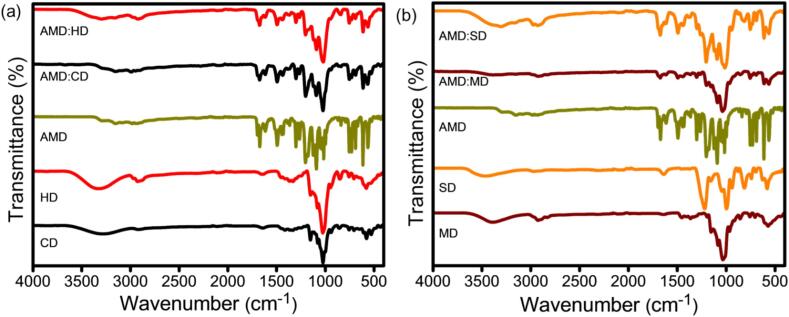
FTIR spectra of (a) CD, HD, AMD, and their IC of AMD:CD and AMD:HD and (b) MD, SD, AMD, and their IC of AMD:MD and AMD:SD.

The FTIR spectra of CD, HD, and AMD, and their ICs of AMD:CD and AMD:HD, are shown in Fig. 4a. The characteristic O—H and C—H stretching frequencies of CD and HD were obtained at 3350 and 3412 cm^−1^ and 2912 and 2928 cm^−1^, respectively. The peaks at 1032, 1012, and 1122 cm^−1^ are indicative of the C—C, C—O, and C-O-C stretching vibrations of the CDs' glycoside bridge (Ficarra et al., 2002). The FTIR spectrum of AMD is characterized by distinct peaks. The bands at 3421 cm^−1^ and 1472 cm^−1^ correspond to N—H stretching and deformation vibrations, respectively. The functional groups C=O, C=C, and C—N show valence vibrations at 1645, 1662, and 1202 cm^−1^, respectively. In addition, bands at 1300 and 1092 cm^−1^ indicate the stretching vibrations of C—O. Moreover, aromatic C=C and C—H vibrations were observed at 1623 cm^−1^ and 734 cm^−1^, respectively. In the FTIR spectra of the ICs of AMD:CD and AMD:HD, the characteristic O—H peak of CD and HD shifted to a lower wavenumber, and the intensities were also reduced. However, the aromatic C=C stretching vibration peak of AMD shifted from 1623 to 1654 cm^−1^ because AMD was hydrophobically complexed with the CD and HD cavities through non-covalent interactions.

For the comparison study, the FTIR spectra of MD, SD, and AMD and their ICs, AMD:MD and AMD:SD, are presented in Fig. 4b. The characteristic aliphatic C—H and the primary and secondary C-OH groups of MD were observed at 2841 and 1040 cm^−1^. In the SD spectrum, the corresponding S=O stretching vibration was indicated by peaks at 1356 and 1134 cm^−1^, whereas the peaks at 1602 and 824 cm^−1^ were ascribed to the stretching vibration of the C=C aromatic group. The ICs of AMD:MD and AMD:SD were not similar to those of AMD, but they were very similar to those of AMD:MD and AMD:SD because the host molecules overlapped with AMD. Furthermore, the C—H and S—H peaks of AMD:MD and AMD:SD were shifted to 2923, 1367, and 1146 cm^−1^ due to the methoxy and sulfur functional groups involved in the complex formation. Moreover, the aromatic ring peak of AMD completely disappeared because it was covered by the MD and SD cavities. Overall, these results indicate that the AMD was included in the selective CD (CD, HD, MD, and SD) cavities (with varying cavity sizes) for IC formation via non-covalent interactions, including hydrogen bonding.

### 3.4 XRD analysis

**Fig. 5:**
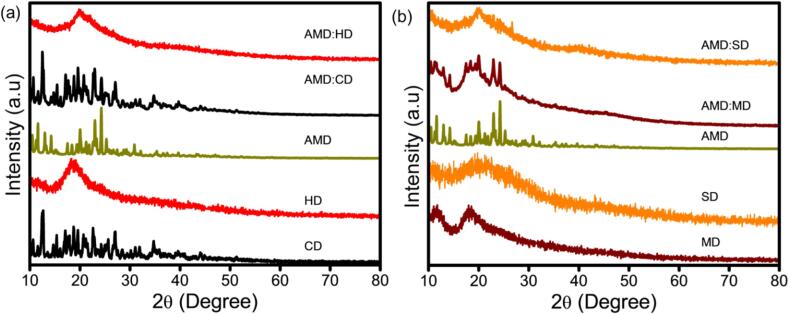
XRD pattern of (a) CD, HD, AMD, and their IC of AMD:CD and AMD:HD and (b) MD, SD, AMD, and their ICs of AMD:MD and AMD:SD.

The XRD technique helps to verify the crystalline or amorphous phases of host–guest complexes. Fig. 5a shows the XRD patterns of CD, HD, and AMD and their ICs of AMD:CD and AMD:HD. In CD, the monoclinic crystalline form was present with 2θ diffraction values of 9.01°, 12.51°, 17.23°, 19.21°, 22.23°, 24.4°, and 35.3° (Wang et al., 2007). The HD pattern confirmed its amorphous nature because of the two broad characteristic peaks of 12.34° and 18.23° (Li et al., 2024). Moreover, AMD is a pure crystalline drug with sharp peaks at 9.43°, 11.23°, 22.21°, and 23.21°. However, when it is converted into a polymer or another form, such as ICs, nanofibers, nanosheets, and films, it forms an amorphous drug (Ghobashy et al., 2020). Some crystalline peaks of AMD were present in the AMD:CD complex; however, the characteristics of CD were more dominant. AMD was included within the CD during the co-precipitation process, which led to a reduction in particle size, although traces of pure AMD were still observed. The XRD pattern of the AMD:HD ICs revealed a broad, large background without AMD crystalline peaks, similar to that of the amorphous HD, thus indicating the formation of a new structure. The lack of crystallinity is a sign that an IC was formed without the presence of residual pure AMD. For a comparison study, the XRD pattern of MD, SD, and AMD and their ICs of AMD:MD and AMD:SD are shown in Fig. 5b. The XRD pattern of MD revealed two broad diffraction peaks of 13.4° and 23.22°, indicating a hollow amorphous state (Trollope et al., 2014), whereas an amorphous broad pattern was obtained for the ICs of SD, which did not exhibit any particular characteristic diffraction peaks (Wang et al., 2004). Thus, after generating the ICs of AMD:MD and AMD:SD, it was shown that reducing the crystalline pattern of the AMD characterization peaks revealed that the distinct patterns of AMD and CDs were roughly superimposed. The lower intensities of the diffraction peaks suggest that the particle sizes decreased during the solid-phase complex preparation. From the IC pattern, the amorphous state loses its crystallinity, indicating that the AMD was covered within the AMD:MD and AMD:SD IC cavities. Overall, these results suggest that the transformation of the XRD pattern corresponds to the amorphization of the AMD molecules after the intramolecular interaction and formation of supramolecular ICs, such as AMD:CD, AMD:HD, AMD:MD, and AMD:SD.

### 3.5 SEM analysis

**Fig. 6:**
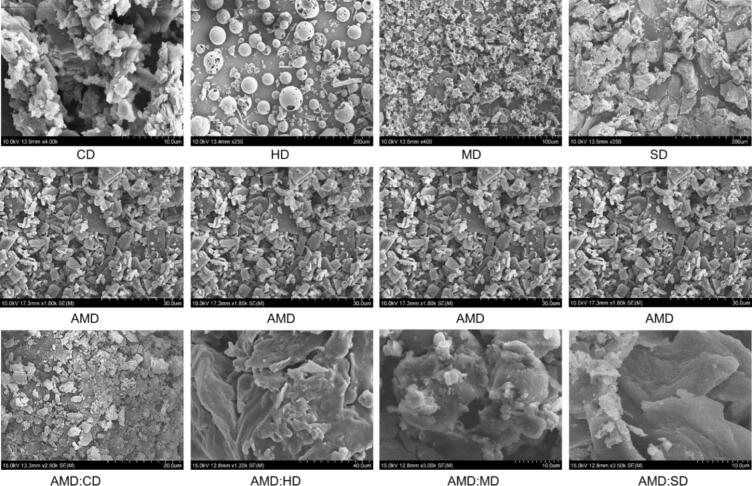
SEM analysis of CD, HD, MD, SD, AMD, and their IC of AMD:CD, AMD:HD, AMD:MD, and AMD:SD.

SEM analysis facilitates the confirmation of IC formation by determining the surface roughness and visualizing the surface texture, shapes, sizes, and structures of the pure and nano-IC particles. The SEM images of CD, HD, MD, SD, and AMD and their ICs of AMD:CD, AMD:HD, AMD:MD, and AMD:SD are shown in Fig. 6. The pure CD exhibited a small crystalline particle, whereas HD exhibited a small spherical structure (Gasbarri and Angelini, 2022; Musuc et al., 2020). An amorphous sphere and an irregular amorphous morphology were observed for MD and SD, respectively (Pardeshi et al., 2023; Ganjali Koli and Fogolari, 2023). The SEM images of AMD revealed a small, crystalline, rod-like morphology, which was consistent with the XRD pattern thereof. In contrast, the SEM images of the AMD:CD, AMD:HD, AMD:MD, and AMD:SD ICs appeared as irregular particles, and the original morphology of the CDs and AMD disappeared. Based on these observations, the material was agglomerated as the AMD drug particles were distributed across the surface of CD, HD, MD, and SD. AMD:SD has more amorphous irregular features compared with those of AMD:CD, AMD:HD, and AMD:MD because of the stronger interaction between AMD and SD, which was previously confirmed in the phase solubility study. Finally, this result elucidates that the aqueous ICs include small particles and homogenous aggregates, which demonstrates the optimal complexation of an amorphous product with a single complex component.

### 3.6 Thermal studies

**Fig. 7:**
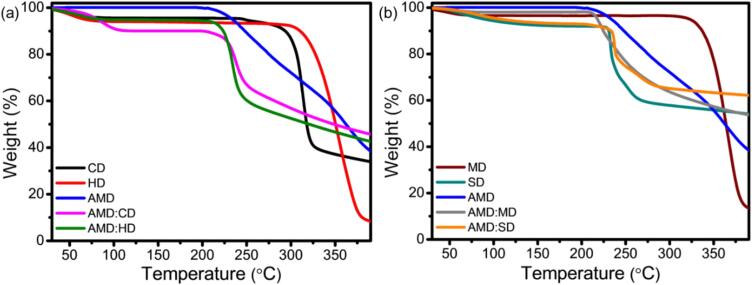
TGA analysis of (a) CD, HD, AMD, and their IC of AMD:CD and AMD:HD and (b) MD, SD, AMD, and their IC of AMD:MD and AMD:SD.

Differences in the thermal stability between the pure AMD and ICs are demonstrated by an experimental TGA thermogram study that provides evidence of the formation of solid ICs between the host and guest molecules (Zhang et al., 2017). CDs have numerous hydroxyl groups that contribute to their thermal resistance (Nie et al., 2022).The TGA thermogram of CD, HD, and AMD, and their ICs of AMD:CD and AMD:HD are shown in Fig. 7a. The pure CDs exhibit two weight loss thermograms because of water loss at 115 °C, and the main degradation was up to approximately 324 °C. As HD is a more labile compound, it undergoes thermal rearrangement at temperatures above 160 °C, and at temperatures above 240 °C, it undergoes thermal decomposition. The AMD drug molecule exhibited moderate thermal stability (almost all residues were decomposed below 220 °C) due to several factors, including photosensitivity and oxidation stress. In addition, the compounds undergo oxidation when exposed to light because the presence of inactive pyridine derivatives influences AMD degradation. The thermal stability of the AMD:CD and AMD:HD complexes was enhanced after the various CD concentrations were fixed at 0.001 M, due to their hydrogen bonding. As a result, the pure AMD degradation peak shifted to higher temperatures of 227 °C and 235 °C because AMD was encapsulated within the CD cavity. In comparison, Fig. 7b exhibited the TGA thermogram of MD, SD, and AMD and their ICs of AMD:MD and AMD:SD. Three weight loss regions were obtained for MD, which are similar to the data reported in the literature. The first one is related to water loss, then the consistent rapid weight loss up to 220 °C was due to partial degradation, and finally, the final weight loss occurrence at 267 °C is due to the complete degradation of MD. In the AMD:MD ICs, the thermal stability was enhanced, which may be because of the increased hydrogen bonding of MD with AMD. Earlier research reported that the methyl groups of the cavity of MD ICs with guest molecules such as drugs, antioxidants, and essential oils provided preferential stability and enhanced the host–guest non-covalent interaction (Zhang et al., 2024). SD exhibited two major weight loss occurrences at 220–250 °C and 300 °C due to desulfonation (SO2) and decomposition of the glucose unit of CD (Wang et al., 2004). In the case of the AMD:SD IC, the desulfonation weight loss shifted to the higher temperature of 260–280 °C because of the hydrogen bonding networks between the sulfur groups in SD and AMD.

**Fig. 8:**
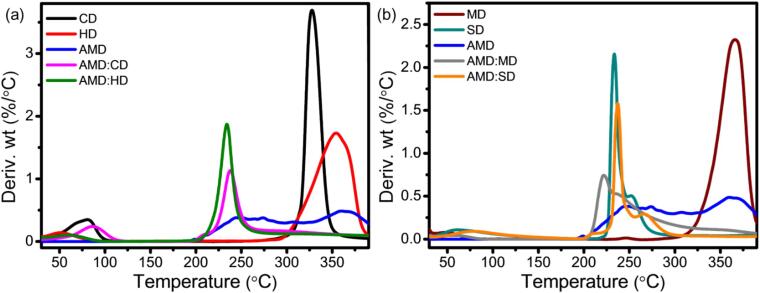
DTG curve of (a) CD, HD, AMD, and their IC of AMD:CD and AMD:HD and (b) MD, SD, AMD, and their and their IC of AMD:MD and AMD:SD.

Fig. 8a represents the derivative thermogravimetric (DTG) curve of the pure molecules and their ICs of AMD:CD, AMD:HD, AMD:MD, and AMD:SD. Here, a little peak at 234 °C was obtained, representing the degradation of AMD, and the same peak was shifted to 248 °C, 245 °C, 248 °C, and 250 °C for AMD:CD, AMD:HD, AMD:MD, and AMD:SD, respectively. Comparatively, the higher thermal stability obtained for AMD:SD may be due to the presence of the sulfur group that helps to retard the effect of increased temperatures. Many researchers have reported that sulfur-containing polymers exhibit enhanced thermal stability, which can form C-H…S interactions (Jiang et al., 2023). Therefore, AMD can be protected and its loss delayed, at relatively high temperatures, through complexation with various CDs. Overall, these results indicate that AMD was successfully encapsulated within the CD cavities (CD, HD, MD, and SD). This inclusion enhances the thermal stability of AMD and the CDs' glucose units. The improvement is primarily attributed to the formation of multiple hydrogen bonds, synergistic hydrogen bonding, and electrostatic interactions, which collectively contribute to the increased thermal stability (Pardeshi et al., 2023).

### 3.7 Computational study

**Fig. 9:**
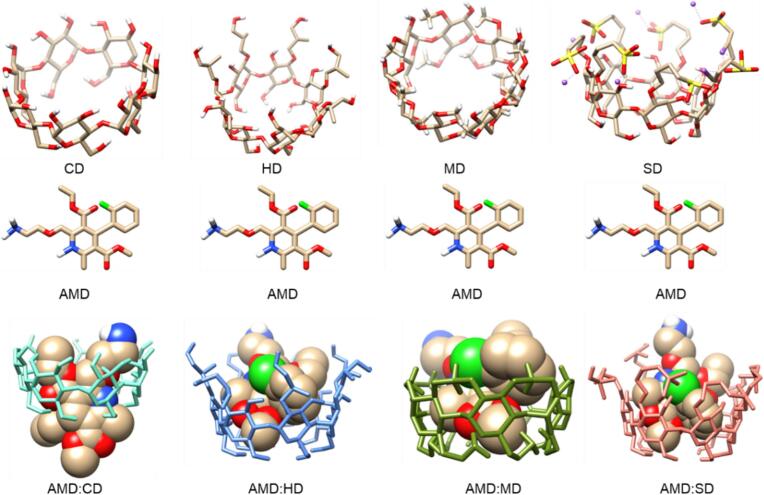
Molecular docking with 3-D structures of pure CDs, and their molecular stimulation diagram of inclusion complex of AMD:CD, AMD:HD, AMD:MD and AMD:SD.

Molecular modeling was performed to provide more information on the complexation process between the supramolecular host and guest compounds. This host–guest interaction indicated that AMD replaced a process in the CDs water molecule cavity through hydrogen bonding or van der Waals forces between the molecules. The most energetically favorable configurations were obtained from the FireDock solutions with the highest global energy, attractive van der Waals forces, and repulsive interactions (Table S-5 and S-6) (Fig. 9). The configuration gives a higher complementarity score, indicating that the ICs are more stable and favorable. Based on this concept, AMD:SD achieved a higher binding score (4840) than that of AMD:CD, AMD:HD, and AMD:MD (4338, 4536, and 4418, respectively). In general, the more negative the atomic contact energy between the host and guest molecules is, the more stable the complexation because it releases energy during complex formation. This means that when the host and guest molecules bond to the lower energy state, the complexes are thermodynamically more stable, which reduces the process of dissociation (Zhang et al., 1997).

The atomic contact energies of the CD:AMD, AMD:HD, AMD:MD, and AMD:SD ICs are −18.60, 17.98, 23.22, and 20.43 kcal/mol, respectively. In addition, the global energy of the complexation values are −47.13, −50.56, −55.36, and − 56.70 for AMD:CD, AMD:HD, AMD:MD, and AMD:SD, respectively. A negative global energy indicates that a more stable complexation is possible because the low negative global energy suggests a significant binding affinity, which also explains the more stable complex and probable binding interaction between them (Mohanty and Mohanty, 2023; Khelfaoui et al., 2021). Furthermore, docking studies can identify specific interactions, such as hydrogen bonds or hydrophobic interactions, that enhance the stability of complexes. The attractive energy values for the ICs of AMD:CD, AMD:HD, AMD:MD, and AMD:SD are −19.56, −19.72, −25.79, and − 20.19 kcal/mol, respectively, while the repulsive energy values are 14.83, 7.46, 25.32, and 5.81 kcal/mol, respectively. The lower repulsive energy and higher attractive energy suggest that hydrogen bonding interactions occurred between AMD and the CDs because of the repulsion caused by the water molecules, which strive to improve their hydrogen bonding while minimizing their interaction with non-polar substances. In general, our theoretical findings of the AMD:CD, AMD:HD, AMD:MD, and AMD:SD ICs warrant further attention because the complexation of AMD is appropriate with all CDs. From our experimental perspective, comparable results were provided in all cases.

### 3.8 Drug release studies

Previous researchers have reported that the formation of ICs is an effective way to delay the release of guest molecules (Jacob and Nair, 2018). These studies aid in clarifying the mechanisms of drug release, such as diffusion, dissociation, and competition with other molecules. However, the specific releasing mechanism can vary depending on the structures of the ICs, preparation methods, and physicochemical characteristics of the guest and host molecules. AMD is slightly soluble in aqueous medium; 2.93 mg dissolves in 1 mL of water at 37 °C (Sheraz et al., 2016). Previously, researchers modified AMD with PEG4000 and PEG6000 using the solid dispersion method, which increased the solubility and bioavailability of the drug (Hamzah et al., 2022). Recent research has focused on the development of supramolecular complexation using CD and HD as host molecules. Investigations have been conducted using phosphate-buffered saline (PBS, pH ∼ 7.4) solution, as it is considered neutral to slightly alkaline, thereby mimicking the physiological pH of blood and colonic fluids in the human body. This characteristic is essential for maintaining stability and biocompatibility, which are critical for the development of oral drug delivery systems.

**Fig. 10:**
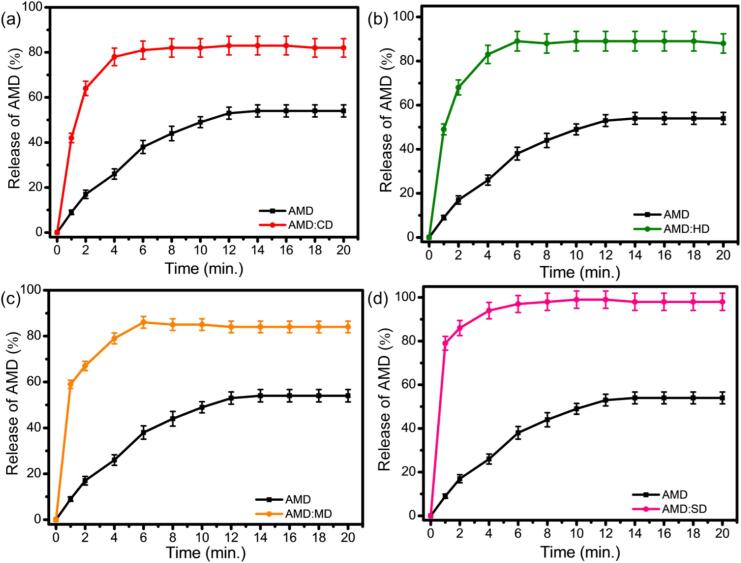
The cumulative release of AMD and its IC of (a) AMD:CD, (b) AMD:HD, (c) AMD:MD, and (d) AMD:SD in aqueous solution.

Fig. 10 shows the cumulative release of AMD and its ICs of AMD:CD, AMD:HD, AMD:MD, and AMD:SD in aqueous solution. Sustained release was observed in all cases, and the release profile of pure AMD was 48 % within 12 min. The release profiles of the ICs exhibited two different stages, including the burst effect called the quick release phase, which occurs within the first 2 min, and the second stage is the slow, plateaued release rate, which indicates that it is controlled. The SD ICs exhibited a better result than the ICs generated with the other CDs, which may be due to the host and guest molecules being tightly bonded; therefore, it is less likely to dissociate. According to a previous phase solubility study, sulfated CDs typically exhibit higher binding constants (k) and solubility profiles because the negatively charged sulfate groups interact strongly with the positively charged AMD molecules. Therefore, the binding strength and solubility directly affect the rate of the AMD release profile. A stronger binding affinity makes it more difficult for the drug to separate from the IC, potentially slowing its release. In contrast, higher solubility promotes faster release, as the complex disperses more easily into the surrounding solution. Furthermore, the complex is more readily reformed after the release of some guest molecules, which may result in a sustained release profile.

Nanda research group conducted a similar investigation, introducing a film-like AMD material with SD that released more than 93 % of the drug over 6 h (Nanda et al., 2018). In contrast, our study yielded superior outcomes, likely because of the formation of a nanopowder AMD:SD ICs, which more effectively reduces the particle size compared to that of film-like materials. In addition, the drug molecule was thoroughly integrated into the CDs amorphous state during nanopowder formation. These elements contribute to a more enhanced release profile compared with what was previously reported. The quick-release phase is considered to be attributed to the free AMD adsorbed onto the particle surface. It requires 2 min to attain the maximum released concentrations of 45 μg/mg, 55 μg/mg, 62 μg/mg, and 83 μg/mg for the AMD:CD, AMD:HD, AMD:MD, and AMD:SD ICs, respectively, and thereafter the active compound is released by diffusion from the CD cavity of the surface. The sustained release percentage of AMD is 82 %, 85 %, 88 %, and 98 % from the AMD:CD, AMD:HD, AMD:MD, and AMD:SD ICs within 20 min, respectively. The drug release profile exhibited an increasing order of AMD:CD < AMD:HD < AMD:MD < AMD:SD.

### 3.9 Anticancer study

**Fig. 11:**
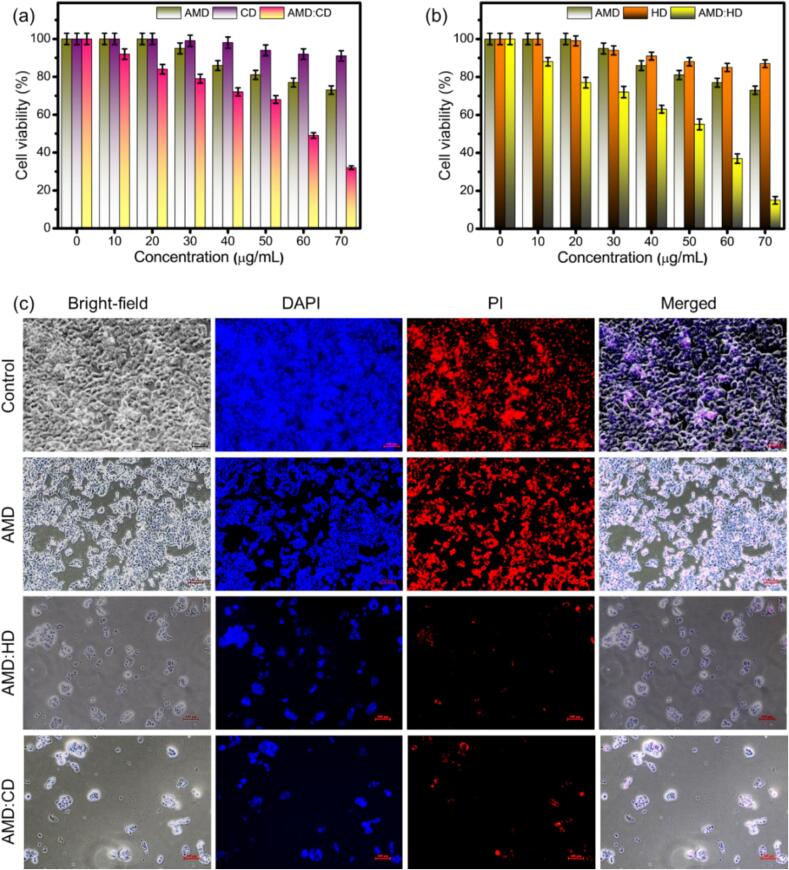
Cell viability of (a) AMD, CD, and AMD:CD (b) AMD, HD, and AMD:HPCD (10–70 μg/mL) on HCT-116 cells after 24 h. (c) Bright-field, DAPI and PI-staining, and merged images of HCT-116 cell lines after incubation for 24 h with control, AMD, AMD:CD, and AMD:HD at 70 μg/mL.

**Fig. 12:**
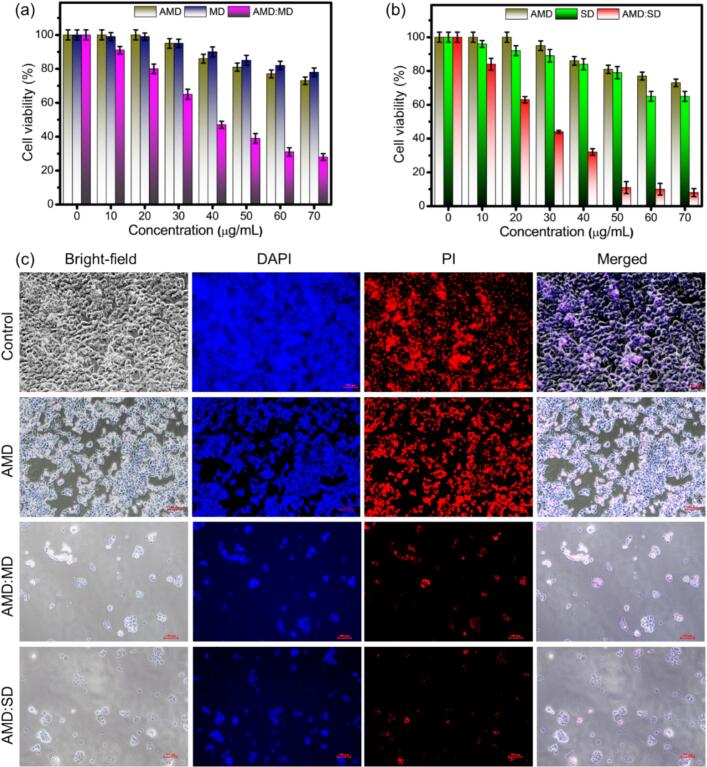
Cell viability of (a) AMD, MD, and AMD:MD (b) AMD, SD, and AMD:SD (10–70 μg/mL) on HCT-116 cells after 24 h. (c) Bright-field, DAPI and PI-staining, and merged images of HCT-116 cell lines after incubation for 24 h with control, AMD, AMD:MD, and AMD:SD at 70 μg/mL. (Scale bar∼100 μm).

AMD and its derivatives are naturally effective anticancer agents against colon cancer cell lines (Alandağ and Öztürk, 2022). Here, we studied the anticancer properties of AMD-incorporated ICs of AMD:CD, AMD:HD, AMD:MD, and AMD:SD in the concentration range of 0–70 μg/mL by determining the cytotoxicity effect against the colon cancer cell line HCT-116 using the WST assay. Moreover, we tested the pure AMD, CD, HD, MD, and SD materials, as shown in Fig. 11, Fig. 12. The increasing concentration of the materials gradually decreased the cell viability (Nanda et al., 2018). Moreover, the cell viability of HCT-116 cells was 78 %, 97 %, 88 %, 82 %, and 78 % when incubated with the pure AMD, CD, HD, MD, and SD, respectively (Fig. 11a, b and 12a, b).

The cell viability of HCT-116 cells gradually decreased when incubated with AMD:CD, AMD:HD, AMD:MD, and AMD:SD at the maximum concentration (70 μg/mL), with values of 25 %, 18 %, 23 % and 10 %, respectively (Fig. 11a, b and 12a, b). Among these, the highest activity was obtained with AMD:SD, which can be attributed to the sulfur functional group that is responsible for the greater complexation efficiency, phase solubility, and bioavailability revealed by our experimental study. It has been reported that the effect on cytotoxicity differs depending on the pure drug and IC formation, and the anticancer cytotoxicity of drugs can be affected by the inclusion of CDs (CD, HD, MD, and SD) as the solubility and bioavailability of the poorly soluble drug are improved, which may lead to improved drug efficacy and reduced toxicity (Zhang et al., 2011a, b).

In addition, the bright-field, DAPI, and PI staining and the merged images of the HCT-116 cell lines after incubation for 24 h with the control, pure molecules, AMD:CD, AMD:HD, AMD:MD, and AMD:SD at 70 μg/mL are shown in Fig. 11c and 12c. It is beneficial to confirm the morphological features of the cell viability of HCT-116 cells incubated with the pure and prepared ICs materials. Based on cell cytotoxicity and the enhanced chemical and biological properties, the use of the produced ICs is suggested for the treatment of human colon cancer.

## 4 Conclusion

In this study, we demonstrated the successful formation of ICs of AMD:CD, AMD:HD, AMD:MD, and AMD:SD in solid (co-precipitation) and solution (PBS; pH ∼ 7.4) medium. The B—H plot confirmed the 1:1 stoichiometry of the ICs, and the binding constant (K_a_) values were 52.74, 87.42, 99.99, and 155.40 M^−1^ for the β-AMD:CD, AMD:HD, AMD:MD, and AMD:SD ICs, respectively. In addition, the phase solubility test suggests that the solubility was enhanced, and the FTIR spectroscopic analysis results indicate that AMD was efficiently incorporated into the CD cavities. The XRD and SEM analyses elucidate that the prepared ICs have amorphous characteristics and are distinctly different from the AMD and CD pure materials. The enhanced thermal stability was confirmed by TGA studies, and the theoretical computation studies demonstrated the establishment of stable IC formations. The sustained release profile was obtained using UV–Vis drug release studies; this exhibited an increasing order of AMD:CD < AMD:HD < AMD:MD < AMD:SD, which confirmed that the release capacity increased when the solubility and stability were increased. Furthermore, the generated AMD ICs exhibited excellent anticancer activity against the HCT-116 colon cancer cell line. Therefore, the ICs of AMD:CD, AMD:HD, AMD:MD, and AMD:SD have the potential as a cancer treatment strategy and may be explored for future clinical applications due to their high solubility, stability, bioavailability, and biological activity.

## CRediT authorship contribution statement

**Sonaimuthu Mohandoss:** Writing – review & editing, Writing – original draft, Supervision, Methodology, Conceptualization. **Kuppu Sakthi Velu:** Methodology, Conceptualization. **Naushad Ahmad:** Methodology, Funding acquisition. **Ramachandran Srinivasan:** Writing – original draft, Methodology. **Prasanta Roy:** Writing – original draft, Validation. **Prathap Somu:** Writing – review & editing, Validation, Conceptualization. **Dibyajyoti Haldar:** Writing – review & editing, Visualization.

## Declaration of competing interest

The authors declare that they have no known competing financial interests or personal relationships that could have appeared to influence the work reported in this paper.

## Data Availability

Data will be made available on request.

## Appendix A Supplementary data

Supplementary material Image 1
