# Supplementary material for: Comparative study of β-cyclodextrin derivatives with amlodipine inclusion complexes for enhanced solubility, drug release, and anticancer activity
Source: Int J Pharm X. 2025 Jul 23;10:100368. doi: 10.1016/j.ijpx.2025.100368 (PMC12320181; doi:10.1016/j.ijpx.2025.100368)
Supplement: Supplementary file 1 — Supplementary material [file mmc1.docx]

**Comparative study of β-cyclodextrin derivatives with amlodipine inclusion complexes for enhanced solubility, drug release, and anticancer activity**

Sonaimuthu Mohandoss^a,b,1,^*, Kuppu Sakthi Velu^b,1^, Naushad Ahmad^c^, Ramachandran Srinivasan^d^, Prasanta Roy^b,1^, Prathap Somu^e,^*, Dibyajyoti Haldar^f,*^

*^a^Centre of Molecular Medicine and Diagnostics, Saveetha Dental College and Hospitals, Saveetha Institute of Medical and Technical Sciences, Saveetha University, Chennai, Tamil Nadu, India*

*^b^School of Chemical Engineering, Yeungnam University, Gyeongsan-38541, Republic of Korea,* [*sakthi.velu4@yu.ac.kr*](mailto:sakthi.velu4@yu.ac.kr) *&* [*prasantaray86@gmail.com*](mailto:prasantaray86@gmail.com)

*^c^Department of Chemistry, College of Science, King Saud University, P.O. Box 2455, Riyadh-11451, Saudi Arabia,* [*anaushad@ksu.edu.sa*](mailto:anaushad@ksu.edu.sa)

*^d^Centre for Ocean Research, Sathyabama Research Park, Sathyabama Institute of Science and Technology, Chennai 600119, Tamil Nadu, India,* [*srinivan.cor@sathyabama.ac.in*](mailto:srinivan.cor@sathyabama.ac.in)

*^e^Department of Biotechnology and Chemical Engineering, School of Engineering, Faculty of Science, Technology and Architecture, Manipal University Jaipur, Dehmi Kalan, Jaipur-Ajmer Expressway, Jaipur- 303007, Rajasthan, India.*

*^f^Department of Biotechnology, Manipal Institute of Technology, Manipal Academy of Higher Education (MAHE), Manipal- 576104, Karnataka, India.*

-------------------------------------------------------------------------

*Corresponding authors:

Sonaimuthu Mohandoss ([drsmohandoss@yu.ac.kr](mailto:drsmohandoss@yu.ac.kr)),

Prathap Somu ([prathap.somu@jaipur.manipal.edu](mailto:prathap.somu@jaipur.manipal.edu)),

Dibyajyoti Haldar ([dibyajyoti.haldar@manipal.edu](mailto:dibyajyoti.haldar@manipal.edu))

^1^ These authors contribute equally to this work.

**Computational method**

Molecular docking is a computational method used to predict the interactions between molecules, typically a ligand (small molecule) and a target receptor (usually a protein). To investigate the mechanism of host-guest inclusion complex formation, the interactions between AMD and β-Cyclodextrin derivatives (CDs; CD, HD, MD, and SD) were studied at the molecular level using molecular docking simulations. The molecular docking of AMD with β-CDs was performed using the PatchDock and FireDock servers. The 3D structures of AMD and the CD derivatives were drawn using ChemDraw Professional 16.0 and Gaussian 9.0, and then optimized using Chem3D 16.0 (CambridgeSoft, PerkinElmer). The structures were saved in PDB format and prepared for input into the PatchDock and FireDock servers. The 3D structures of AMD and the CDs were uploaded to the PatchDock server (http://bioinfo3d.cs.tau.ac.il/PatchDock/) for initial docking followed by refinement using the FireDock server (http://bioinfo3d.cs.tau.ac.il/FireDock/). The docking complexes were then downloaded from FireDock after refinement. The results were visualized and analyzed using Chimera, a molecular visualization tool. Finally, using the default scoring mechanisms of PatchDock and FireDock, the configuration with the highest docking score and the lowest binding affinity (kcal/mol) was selected as the optimal interaction model.

**Table S-1.** Absorption and fluorescence spectral maxima of AMD with various concentrations of CD at pH 7.4.

| CD concentration | Abs | FL. Int. | [1/CD] | [1/A-A_0_] | [1/F-F_0_] |
| --- | --- | --- | --- | --- | --- |
| 0 | 0.171 | 186 | 0 | 0 | 0 |
| 0.002 | 0.322 | 207 | 500 | 6.6225 | 0.0481 |
| 0.004 | 0.418 | 229 | 250 | 4.0485 | 0.0232 |
| 0.006 | 0.514 | 246 | 166.66 | 2.9154 | 0.0167 |
| 0.008 | 0.611 | 267 | 125 | 2.2727 | 0.0124 |
| 0.010 | 0.717 | 278 | 100 | 1.8315 | 0.0108 |
| 0.012 | 0.911 | 294 | 83.33 | 1.3531 | 0.0092 |
| Binding constant (K) M^-1^ | | | | 48.30 | 52.74 |
| Gibbs free energy (ΔG) kJ/mol | | | | -9.76 | -9.98 |

**Table S-2.** Absorption and fluorescence spectral maxima of AMD with various concentrations of HD at pH 7.4.

| HD concentration | Abs | FL. Int. | [1/HD] | [1/A-A_0_] | [1/F-F_0_] |
| --- | --- | --- | --- | --- | --- |
| 0 | 0.171 | 186 | 0 | 0 | 0 |
| 0.002 | 0.291 | 240 | 500 | 8.4033 | 0.0185 |
| 0.004 | 0.554 | 288 | 250 | 2.6109 | 0.0097 |
| 0.006 | 0.697 | 331 | 166.66 | 1.9011 | 0.0069 |
| 0.008 | 0.893 | 376 | 125 | 1.3850 | 0.0052 |
| 0.010 | 1.186 | 439 | 100 | 0.9852 | 0.0039 |
| 0.012 | 1.296 | 501 | 83.33 | 0.8888 | 0.0031 |
| Binding constant (K) M^-1^ | | | | 84.27 | 87.42 |
| Gibbs free energy (ΔG) kJ/mol | | | | -11.16 | -11.26 |

**Table S-3.** Absorption and fluorescence spectral maxima of AMD with various concentrations of MD at pH 7.4.

| MD concentration | Abs | FL. Int. | [1/MD] | [1/A-A_0_] | [1/F-F_0_] |
| --- | --- | --- | --- | --- | --- |
| 0 | 0.171 | 186 | 0 | 0 | 0 |
| 0.002 | 0.411 | 238 | 500 | 4.166 | 0.0192 |
| 0.004 | 0.594 | 271 | 250 | 2.364 | 0.0118 |
| 0.006 | 0.769 | 308 | 166.66 | 1.672 | 0.0081 |
| 0.008 | 1.034 | 344 | 125 | 1.158 | 0.0063 |
| 0.010 | 1.266 | 360 | 100 | 0.913 | 0.0057 |
| 0.012 | 1.618 | 375 | 83.33 | 0.691 | 0.0053 |
| Binding constant (K) M^-1^ | | | | 88.70 | 99.99 |
| Gibbs free energy (ΔG) kJ/mol | | | | -11.29 | -11.61 |

**Table S-4.** Absorption and fluorescence spectral maxima of AMD with various concentrations of SD at pH 7.4.

| SD concentration | Abs | FL. Int. | [1/SD] | [1/A-A_0_] | [1/F-F_0_] |
| --- | --- | --- | --- | --- | --- |
| 0 | 0.171 | 186 | 0 | 0 | 0 |
| 0.002 | 0.477 | 222 | 500 | 3.267 | 0.2805 |
| 0.004 | 0.698 | 303 | 250 | 1.897 | 0.0085 |
| 0.006 | 0.913 | 376 | 166.66 | 1.347 | 0.0052 |
| 0.008 | 1.145 |  | 125 | 1.026 | 0.0037 |
| 0.010 | 1.521 |  | 100 | 0.740 | 0.0028 |
| 0.012 | 1.879 |  | 83.33 | 0.585 | 0.0022 |
| Binding constant (K) M^-1^ | | | | 110.76 | 155.40 |
| Gibbs free energy (ΔG) kJ/mol | | | | -11.85 | -12.71 |

**Table S-5.** Molecular docking studies using PatchDock and FireDock servers scores for the top 5 docked models of AMD:CD and AMD:HD inclusion complexes.

|  | PatchDock server | | | FireDock server | | | |
| --- | --- | --- | --- | --- | --- | --- | --- |
| S.  No. | Score | Area  (Å^2^) | ACE (kcal/mol) | Global  Energy  (kcal/mol) | Attractive  VdW  (kcal/mol) | Repulsive  VdW  (kcal/mol) | ACE (kcal/mol) |
| *AMD:CD* | | | | | | | |
| 1 | 4338 | 539.20 | -405.07 | -47.13 | -19.56 | 14.83 | -18.60 |
| 2 | 4336 | 550.50 | -404.84 | -44.00 | -19.32 | 18.37 | -18.61 |
| 3 | 433 | 548.30 | -393.61 | -42.46 | -20.29 | 21.62 | -18.46 |
| 4 | 4292 | 544.80 | -395.32 | -41.63 | -16.45 | 8.92 | -15.54 |
| 5 | 4272 | 541.40 | -395.23 | -33.89 | -17.71 | 24.93 | -17.17 |
| *AMD:HD* | | | | | | | |
| 1 | 4586 | 548.10 | -357.26 | -50.56 | -19.72 | 7.46 | -17.98 |
| 2 | 4268 | 526.20 | -330.13 | -50.07 | -20.36 | 9.82 | -18.25 |
| 3 | 4262 | 513.00 | -386.85 | -41.37 | -17.31 | 8.65 | -15.41 |
| 4 | 4096 | 485.00 | -331.22 | -40.37 | -16.94 | 5.88 | -15.74 |
| 5 | 4064 | 447.10 | -240.24 | -34.24 | -14.04 | 5.72 | -12.65 |

**Table S-6.** Molecular docking studies using PatchDock and FireDock servers scores for the top 5 docked models of AMD:MD and AMD:SD inclusion complexes.

|  | PatchDock server | | | FireDock server | | | |
| --- | --- | --- | --- | --- | --- | --- | --- |
| S.  No. | Score | Area  (Å^2^) | ACE (kcal/mol) | Global  Energy  (kcal/mol) | Attractive  VdW  (kcal/mol) | Repulsive  VdW  (kcal/mol) | ACE (kcal/mol) |
| *AMD:MD* | | | | | | | |
| 1 | 4418 | 605.30 | -530.15 | -55.36 | -25.79 | 25.32 | -23.22 |
| 2 | 4388 | 546.90 | -449.69 | -51.30 | -25.15 | 28.64 | -22.91 |
| 3 | 4358 | 529.60 | -481.25 | -47.85 | -18.50 | 6.69 | -17.18 |
| 4 | 4252 | 527.90 | -434.44 | -47.56 | -22.21 | 13.73 | -18.00 |
| 5 | 4244 | 505.10 | -431.80 | -31.58 | -12.84 | 8.04 | -12.88 |
| *AMD:SD* | | | | | | | |
| 1 | 4840 | 572.40 | -464.46 | -56.70 | -20.19 | 5.81 | -20.43 |
| 2 | 4786 | 572.50 | -449.62 | -54.95 | -21.20 | 13.13 | -21.36 |
| 3 | 4584 | 536.50 | -436.20 | -53.80 | -20.74 | 13.36 | -21.00 |
| 4 | 4444 | 498.40 | -332.25 | -52.65 | -17.90 | 8.09 | -20.76 |
| 5 | 4426 | 507.90 | -340.76 | -50.19 | -19.81 | 13.93 | -20.36 |
